# Supplementary figures and images for: Identification of quantitative trait loci (QTLs) regulating leaf SPAD value and trichome density in mungbean (Vigna radiata L.) using genotyping-by-sequencing (GBS) approach
Source: PeerJ. 2024 Feb 21;12:e16722. doi: 10.7717/peerj.16722 (PMC10893866; doi:10.7717/peerj.16722)

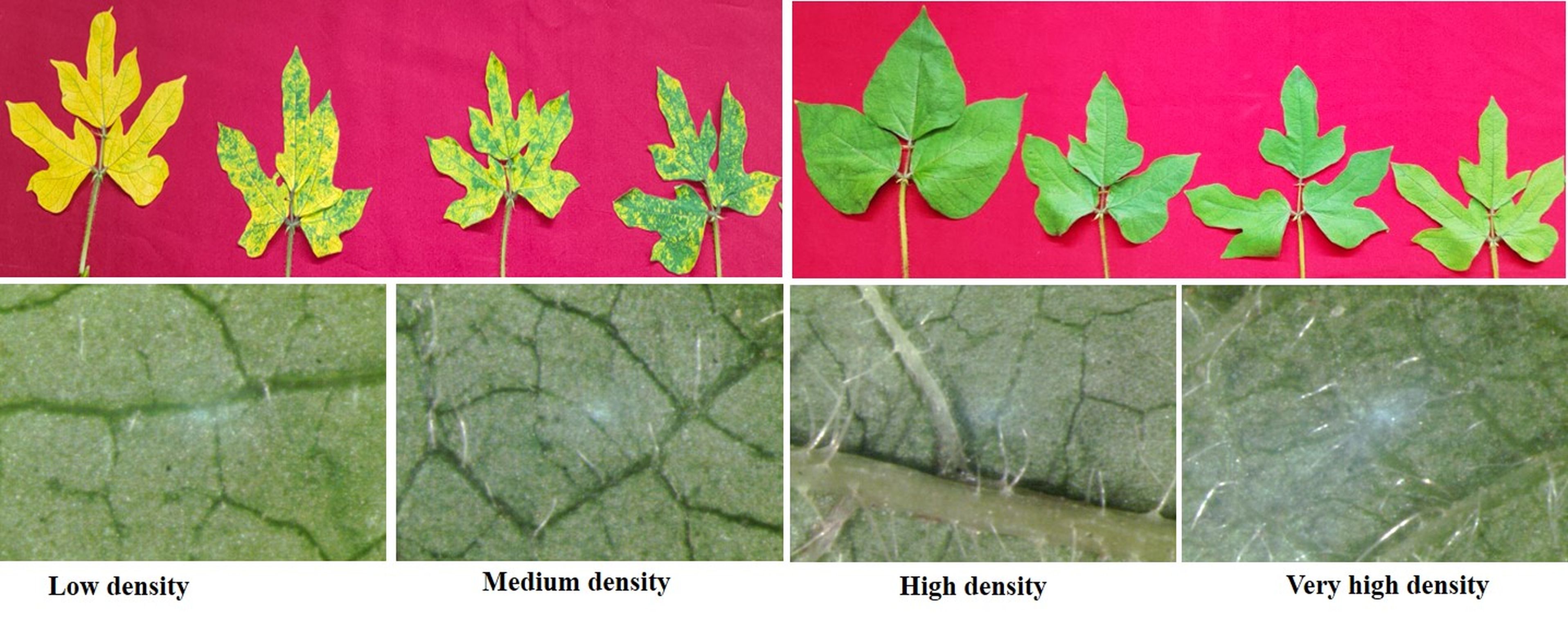

Supplement: Figure S1 [file peerj-12-16722-s003.jpg]

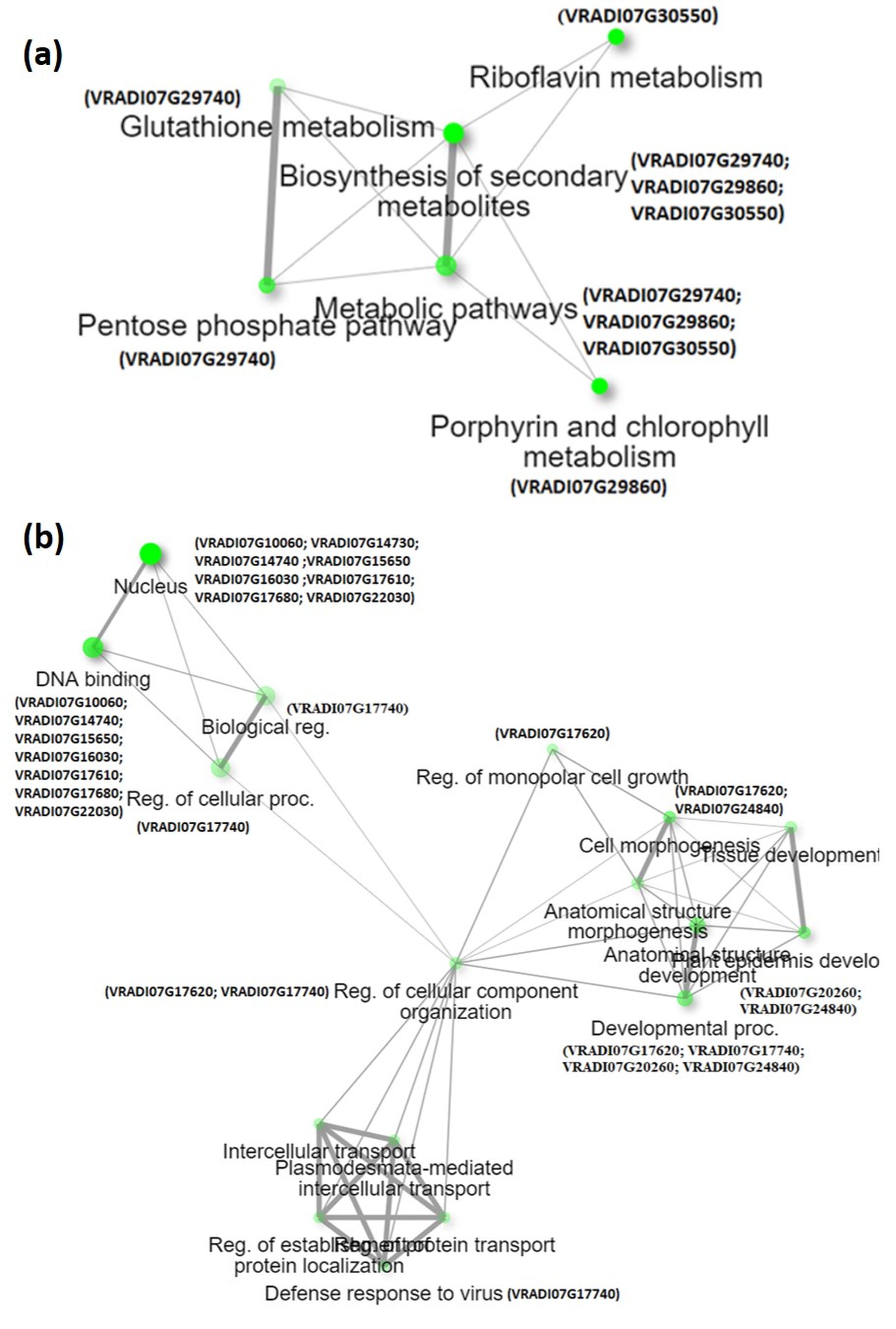

Supplement: Figure S2 [file peerj-12-16722-s004.jpg]
